# A Case of Dual Endocrinopathies Induced by Pembrolizumab Following Treatment for Non-small Cell Lung Cancer: Type 1 Diabetes and Hyperthyroidism/Autoimmune Thyroiditis

**DOI:** 10.7759/cureus.115106

**Published:** 2026-08-24

**Authors:** Carolina Rodriguez, Henry Li, Ankita Ansal, Ihunanya Agomuoh, Kanika Goyal, Geetha Krishnamoorthy, Wael Taha

**Affiliations:** 1 Surgery, Ross University School of Medicine, Bridgetown, BRB; 2 Medicine, Wayne State University School of Medicine, Pontiac, USA; 3 Pulmonology, Ross University School of Medicine, Bridgetown, BRB; 4 Internal Medicine, Trinity Health Oakland/Wayne State University, Pontiac, USA; 5 Internal Medicine, Trinity Health Oakland Hospital, Pontiac, USA; 6 Endocrinology, Wayne State University School of Medicine, Detroit, USA; 7 Endocrinology, Detroit Medical Center, Detroit, USA

**Keywords:** endocrinopathy, hyperthyroidism, immune checkpoint inhibitors, new-onset diabetes, new-onset hyperthyroidism, pembrolizumab, pembrolizumab-induced diabetes mellitus, pembrolizumab-induced thyroiditis, pembrolizumab side effect, programmed cell death ligand 1

## Abstract

Pembrolizumab, a programmed cell death protein 1 (PD-1) immune checkpoint inhibitor, is commonly used in the treatment of squamous cell carcinoma, offering improved survival and tumor response. However, it can rarely lead to immune-related adverse events (irAEs), some of which may be significant. We report a 62-year-old male with squamous cell carcinoma of the lung, a 40-pack-year smoking history, prediabetes, and coronary artery disease, who presented with polydipsia, polyuria, and weakness. He was diagnosed with sudden-onset diabetic ketoacidosis. Laboratory workup revealed negative glutamic acid decarboxylase (GAD), IA-2, and islet cell antibodies, as well as an undetectable C-peptide level, consistent with immune checkpoint inhibitor-induced type 1 diabetes. Concurrently, the patient developed hyperthyroidism with positive thyroid peroxidase (TPO) antibodies, suggesting autoimmune thyroiditis. This case underscores the importance of baseline screening and ongoing monitoring for endocrine irAEs during immunotherapy. Clinicians should maintain a high index of suspicion, even in patients without prior autoimmune disorders. Early recognition, patient education, and coordinated multidisciplinary care are critical for reducing complications and improving outcomes.

## Introduction

Pembrolizumab, an immune checkpoint inhibitor (ICI), was granted breakthrough therapy designation for advanced multiple myeloma in 2014, with the same designation extended to non-small cell lung cancer the following year [1]. As a humanized monoclonal IgG4 antibody, pembrolizumab functions by targeting the programmed cell death protein 1 (PD-1) receptor on the surface of neoplastic cells [2-4]. PD-1 and programmed cell death ligand 1 (PD-L1) are proteins normally expressed on the cell membrane of immune cells and work to regulate the activation of self-tolerance within the immune system [4-6]. The binding of PD-1 to PD-L1 results in the prevention of the autoactivation of the adaptive immune system [4]. Thus, any inhibition of the association of PD-1 to PD-L1 results in an increased autoimmune response mediated by the adaptive immune system and subsequent apoptosis. Reciprocally, in the case of various neoplastic cells, the increased presence of PD-1 on the surface membranes results in a decreased ability for the adaptive immune system to recognize the neoplastic cells, increasing survival and proliferation of the tumor. Pembrolizumab directly blocks PD-1 from accepting its ligand, allowing the immune system to continue to recognize and effectively attack cancerous cells through a T-cell-mediated anti-tumor immune response [6,7]. Antineoplastic therapies that inhibit immune checkpoints have become an established option in cancer treatment. Pembrolizumab, a first-line monotherapy, has been shown to improve progression-free and overall survival in patients with PD-L1-expressing advanced or metastatic non-small cell lung cancer.

With greater longevity comes a higher likelihood of incurring immune-related adverse events (irAEs). IrAEs are manifested by the same immunologic modulation that drives the therapeutic effects of treatment in off-target receptor populations. The Keynote-042 study published in the spring of 2019 observed adverse events of grade 3 or worse in 18% of their patients treated with pembrolizumab compared with 41% in the chemotherapy group [8]. Grade 3 adverse events are serious enough to interfere with one's ability to perform activities of daily living [9]. IrAEs most frequently observed include non-specific activation within the skin, endocrine, lung, gut, hepatic, and musculoskeletal systems. In a cohort study of 387 patients, 43% developed a chronic irAE [10]. The studies also observed an increase in median progression-free survival and overall survival in those who had ≥2 irAEs compared with those with ≤1 irAEs [2]. While this may bode well for patients’ prognosis, acute emergencies of rare presentations increase mortality risk as well as call for thoughtful case management.

## Case presentation

Our patient was a 62-year-old male whose routine lung cancer screening revealed a mass in the left lower lobe of the lung. Biopsy identified the mass as a T3 N2 squamous cell carcinoma with metastasis to the mediastinal lymph nodes. Medical history included 40 pack-years of smoking, prediabetes (glycosylated hemoglobin (HbA1c) = 5.8%), coronary artery disease, hyperlipidemia, and migraines. There was no personal or family history of endocrine or autoimmune disorders.

The patient was on his 4th cycle of neoadjuvant chemotherapy per the KEYNOTE-671 trial [3]: four cycles of Gemzar, cisplatin, and pembrolizumab, followed by 13 cycles of pembrolizumab maintenance following surgery. Plans for surgical resection were interrupted when the patient presented to the emergency department with generalized weakness, polyuria, polydipsia, and excessive vomiting two weeks after the last chemotherapy dose and 11 weeks from the initiation of neoadjuvant chemotherapy. He denied fever, chills, chest pain, shortness of breath, vision changes, diarrhea, constipation, or paresthesia.

At presentation, he was afebrile, with a heart rate of 88 beats per minute, a respiratory rate of 20 breaths per minute with use of accessory muscles, blood pressure of 110/59 mmHg, and oxygen saturation of 79% on room air. Examination revealed dry oral mucosa. No thyroid nodules were detected during the examination. The remainder of the exam was unremarkable.

**Figure 1:**
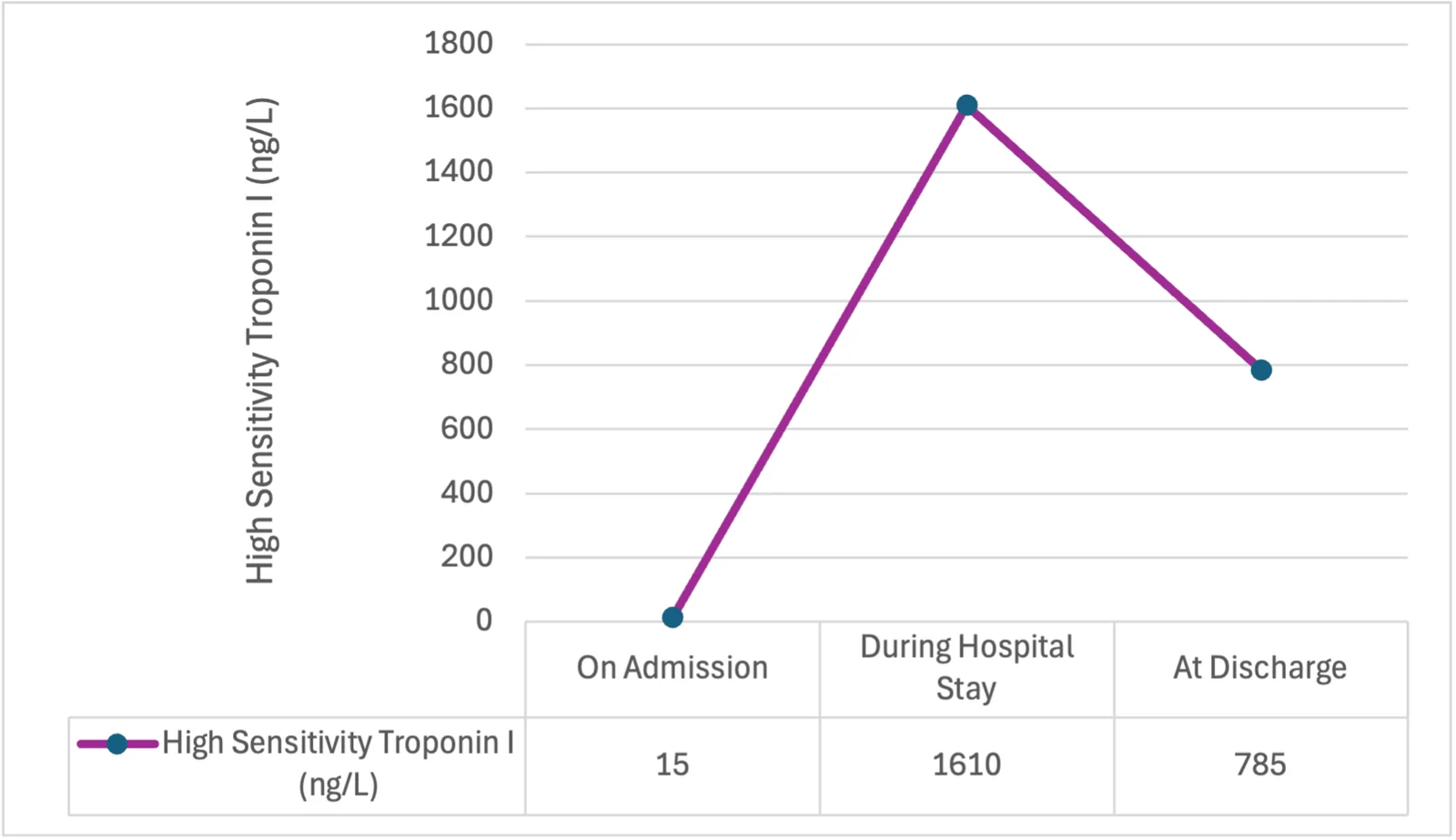
Changes in high-sensitivity troponin I from admission to discharge.

Pertinent laboratory findings showed a blood glucose level of 1,150 mg/dL, phosphorus of 10.6 mg/dL, uric acid of 8.6 mg/dL, beta-hydroxybutyrate >8.00 mmol/L, lipase of 95 U/L, sodium of 128 mmol/L, potassium of 7.4 mmol/L, bicarbonate of 9 mmol/L, anion gap of 34, blood urea nitrogen (BUN) of 62 mg/dL, creatinine of 2.59 mg/dL, blood osmolality of 374 mOsm/kg, urine glucose ≥1,000 mg/dL, urine ketones of 1 mg/dL, and lactate level of 6 mmol/L, as shown in Table 1. High-sensitivity troponin levels were 785 ng/L, increasing to a high of 1631 ng/L, as shown in Figure 1.

**Table 1:** Summary of laboratory findings. LDH: lactate dehydrogenase; ALT: alanine aminotransferase; SGPT: serum glutamic-pyruvic transaminase; AST: aspartate aminotransferase; SGOT: serum glutamic-oxaloacetic transaminase; A/G ratio: albumin-to-globulin ratio; BUN: blood urea nitrogen; GFR: glomerular filtration rate; POCT: point-of-care testing.

Labs	Values	Reference ranges
Phosphorus	10.6 mg/dL	2.4-4.6 mg/dL
Procalcitonin	0.65 ng/mL	<=0.50 ng/mL
Uric acid	8.6 mg/dL	4.4-7.6 mg/dL
LDH	242 unit/L	140-271 unit/L
Beta-hydroxybutyrate	>8.00 mmol/L	0.02-0.27 mmol/L
Magnesium	2.7 mg/dL	1.7-2.5 mg/dL
Lipase	95 unit/L	11-82 unit/L
ALT (SGPT)	19 unit/L	7-52 unit/L
AST (SGOT)	16 unit/L	13-39 unit/L
Alkaline phosphatase	123 unit/L	27-120 unit/L
Bilirubin, direct	0.1 mg/dL	0.0-0.2 mg/dL
Total bilirubin	0.4 mg/dL	0.3-1.2 mg/dL
Total protein	6.8 g/dL	6.1-7.9 g/dL
Albumin	4.5 g/dL	3.5-5.7 g/dL
Globulin, total	2.3 g/dL	2.3-3.5 g/dL
A/G ratio	2	1.1-2.2
Sodium	128 mmol/L	135-144 mmol/L
Potassium	7.4 mmol/L	3.5-5.3 mmol/L
Chloride	85 mmol/L	98-107 mmol/L
CO2	9 mmol/L	21-31 mmol/L
Anion gap	34	3-11
Glucose	1,150 mg/dL	70-99 mg/dL
BUN	62 mg/dL	7-25 mg/dL
Creatinine	2.59 mg/dL	0.70-1.30 mg/dL
Calculated GFR	27 mL/min/1.73 m^2^, >=60 mL/min/1.73 m^2^	
Glucose POCT	>550 mg/dL	70-99 mg/dL
Blood osmolality	374 mOsm/kg	275-295 mOsm/kg
Beta-hydroxybutyrate	>8 mmol	0.02-0.27 mmol/L
Glucose, urine	>=1000 mg/dL	Negative
Ketones, urine	1 mg/dL	Negative
Lactate	6 mmol/L	0.5-2 mmol/L
Troponin	1495 ng/L	<=20 ng/L
C-peptide	0.8-3.9 ng/mL	0.1 ng/mL
Insulin antibody	<0.4 U/mL	0.0-0.4 U/mL
IA-2 antibody	<5.4 IU/mL	<5.4 IU/mL
Islet cell antibody IgG	<1:4	<1:4
Glutamic acid decarboxylase antibody	<5 IU/mL	<5 IU/mL
Thyroid-stimulating immunoglobulin	<0.10 IU/L	<0.10 IU/L
Thyroid peroxidase (TPO) antibody	12 IU/mL	<9 IU/mL
Free T4	1.78 ng/mL	0.61-1.24 ng/mL
Thyrotropin antibody	12 IU/ml	<9 IU/ml

The patient was started on intravenous lactated Ringer's solution and an insulin drip for diabetic ketoacidosis (DKA) and acute kidney injury (AKI). Due to the patient's condition, an ECG was performed, along with a cardiology consult, resulting in a non-ST-elevation myocardial infarction, type 2. The patient was given heparin IV for 48 hours, along with dual antiplatelet therapy, and was continued on ezetimibe, bempedoic acid, and beta blockers. Tamsulosin 0.4 mg was continued for benign prostatic hyperplasia, and topiramate for migraines. DKA and AKI were resolved, and the patient was transferred to subcutaneous glargine and fast-acting lispro insulin with meals. The patient was started on methimazole pending confirmation of thyrotoxicosis etiology. The patient was advised to follow up with an endocrinologist for management of type 1 diabetes mellitus.

## Discussion

ICIs, particularly PD-1 inhibitors like pembrolizumab, have revolutionized the treatment of advanced cancers such as non-small cell lung cancer. However, their mechanism of immune activation can breach self-tolerance and trigger a wide range of irAEs, including endocrinopathies. While isolated irAEs are known to occur with PD-1 inhibitors, the presence of multiple endocrinopathies induced by pembrolizumab is exceedingly rare [11]. Our patient developed two such irAEs: DKA secondary to new-onset type 1 diabetes mellitus and hyperthyroidism due to autoimmune thyroiditis, following multiple cycles of pembrolizumab.

Although rare, ICI-induced type 1 diabetes is a serious and potentially life-threatening complication. The onset is often sudden and severe, typically presenting as DKA, as seen in our patient [12]. In contrast to classic autoimmune diabetes, many patients exhibit low or undetectable levels of pancreatic autoantibodies, and the fulminant presentation suggests rapid β-cell destruction [12]. Our patient tested negative for glutamic acid decarboxylase (GAD), IA-2, and islet cell antibodies, consistent with reports that 50-70% of ICI-induced diabetes cases lack these markers. Notably, his C-peptide level was undetectable, confirming insulin deficiency [12,13]. Pembrolizumab has been shown to trigger hyperglycemia in up to 49% of patients, with grade 3/4 hyperglycemic events occurring in 3-6% and DKA in approximately 0.7-1% of cases [13].

Concurrent endocrine toxicities are uncommon but have been documented. In our patient, hyperthyroidism with positive thyroid peroxidase (TPO) antibodies suggests autoimmune thyroiditis. This aligns with data showing that thyroid dysfunction, particularly painless thyroiditis or transient hyperthyroidism, affects approximately 15% of patients receiving PD-1 inhibitors [13]. Destructive thyroiditis is considered the most common thyroid irAE [14]. Importantly, routine thyroid function monitoring is recommended during ICI therapy due to the variable timing and often asymptomatic nature of onset [13,15].

The overlap of multiple endocrinopathies in the absence of personal or family history argues against genetic predisposition. Anti-PD-1 agents have been implicated in triggering autoimmune polyendocrine syndrome type 2 (APS-2), particularly in individuals with the human leukocyte antigen DR4 (HLA-DR4) haplotype [15]. Although spontaneous APS-2 typically presents in the third or fourth decade of life, immune checkpoint blockade appears to lower this threshold or reveal latent susceptibility. A literature review found that 63% of patients with anti-PD-1-associated APS-2 were HLA-DR4-positive, compared with 35.2% in spontaneous APS-2, supporting a role for genetic screening in high-risk populations [12]. While human leukocyte antigen (HLA) typing is not currently routine, it may serve as a useful biomarker for risk stratification if validated in larger studies [15].

The first-line treatment for most irAEs is high-dose corticosteroids, which aim to suppress the immune response. However, in the case of ICI-induced type 1 diabetes, steroid therapy has not shown benefit in reversing β-cell destruction [12,16]. Insulin remains the mainstay of treatment for hyperglycemia and DKA, in accordance with established protocols for DKA [13]. For thyrotoxicosis, glucocorticoids and anti-thyroid medications are appropriate if hyperthyroidism is significant until the disease etiology is confirmed. Autoimmune thyroiditis is more common after pembrolizumab; however, Graves’ disease is possible. Glucocorticoids and beta blockers may be considered for mild cases of symptomatic thyrotoxicosis [14]. Thyroid uptake and scan may be done to confirm the etiology.

Our case supports current recommendations from the Society for Immunotherapy of Cancer and the European Society for Medical Oncology (ESMO), which advocate for baseline screening and routine monitoring of blood glucose, thyroid-stimulating hormone (TSH), free T4, adrenocorticotropic hormone (ACTH), cortisol, and HbA1c during immunotherapy [13,15]. Given the early onset of hyperglycemia in our patient, detected just 11 weeks after initiation of therapy and within two weeks of his last cycle, this case illustrates the critical value of proactive glucose monitoring.

## Conclusions

As ICIs become increasingly integrated into oncology treatment protocols, clinicians must remain vigilant for potential irAEs, even in patients with no prior autoimmune history. The simultaneous development of type 1 diabetes and autoimmune thyroiditis in our patient exemplifies the unpredictable and severe nature of immune-related endocrinopathies. This case highlights the importance of early recognition, patient education, and multidisciplinary coordination in mitigating complications and enhancing outcomes.
